# *In Vivo* Monitoring for Regional Changes of Metabotropic Glutamate Receptor Subtype 1 (mGluR1) in Pilocarpine-Induced Epileptic Rat Brain by Small-Animal PET

**DOI:** 10.1038/s41598-017-15015-2

**Published:** 2017-11-02

**Authors:** Tomoteru Yamasaki, Masayuki Fujinaga, Wakana Mori, Yiding Zhang, Hidekatsu Wakizaka, Nobuki Nengaki, Lin Xie, Akiko Hatori, Ming-Rong Zhang

**Affiliations:** 1 Department of Radiopharmaceuticals Development, National Institute of Radiological Sciences, National Institutes for Quantum and Radiological Science and Technology, 4-9-1 Anagawa, Inage-ku, Chiba, 263-8555 Japan; 2 Department of Radiation Measurement and Dose Assessment, National Institute of Radiological Sciences, National Institutes for Quantum and Radiological Science and Technology, 4-9-1 Anagawa, Inage-ku, Chiba, 263-8555 Japan; 3 SHI Accelerator Service Ltd, 1-17-6 Osaki, Shinagawa-ku, Tokyo, 141-0032 Japan

## Abstract

Metabotropic glutamate receptor subtype 1 (mGluR1) is a crucial pharmacological target for several central nervous system disorders. In this study, we aimed to monitor *in vivo* regional changes of mGluR1 related to neuroinflammation in the brains of rats after pilocarpine-induced status epilepticus (PISE) using longitudinal positron emission tomography (PET). PISE was induced in rats by administering lithium chloride, followed by repeated pilocarpine hydrochloride treatments. PET assessments were conducted using *N*-[4-[6-(isopropylamino)-pyrimidin-4-yl]-1,3-thiazol-2-yl]-*N*-methyl-4-[^11^C]methylbenzamide ([^11^C]ITDM), a selective radioligand for mGluR1, and *N*-benzyl-*N*-[^11^C]methyl-2-(7-methyl-8-oxo-2-phenyl-7,8-dihydro-9H-purin-9-yl)acetamide ([^11^C]DAC), a selective translocator protein PET ligand for neuroinflammation monitoring. PET scans were conducted on PISE rats at 1 day (acute), 1 week (subacute) and 3 weeks (chronic) after repeated seizures. PET with [^11^C]ITDM showed significant decreases of mGluR1 availability (BP_ND_) in the thalamus and hippocampus after PISE over the chronic period. Conversely, PET with [^11^C]DAC exhibited a significant increase of radioactive uptake in the forebrain after the acute period, especially in the thalamus. These conflicting changes in the thalamus indicated negative correlation. In conclusion, PET with [^11^C]ITDM could successfully visualize hippocampal and thalamic declines of mGluR1 related to neuroinflammation, which would help further understanding for mGluR1 functions in neuroexcitotoxicity.

## Introduction

Glutamate is a major excitatory neurotransmitter in the mammalian central nervous system (CNS). Excitatory neurotransmission via glutamate is regulated by receptors, which are classified as ionotropic or metabotropic. Metabotropic glutamate receptors (mGluRs) are G-protein coupled receptors. These are divided into three groups, including eight subtypes (mGluR1–8) based on sequence homology, intracellular transduction pathways and pharmacological properties^1^. Among the eight subtypes, mGluR1 and mGluR5 (belonging to group I) stimulate polyphosphoinositide hydrolysis via activation of phospholipase C. This induces the production of second messengers such as inositol 1,4,5-triphosphate and diacylglyceol^2^. Subsequently, these messengers trigger intracellular calcium release, which can activate protein kinase C involved in activation of transcription and neuroinflammation^3,4^.

Epilepsy is broadly characterized by aberrant neuronal excitability. Various studies have elucidated the role of glutamate in seizures and epilepsy. Seizures induce elevations in extracellular glutamate, which then contribute to neurotoxic damage by activating glutamate receptors. Moreover, repeated seizures can alter the expression of glutamate receptors in neurons, further contributing to epileptogenesis^5^. Thus, glutamate receptors are crucial imaging targets for further understanding of neuroexcitotoxicity mechanisms in epilepsy.

A recent pilot positron emission tomography (PET) imaging study has been performed using [^11^C]ABP688, a widely used PET ligand for mGluR5 in preclinical and clinical studies^6,7^, in rats after pilocarpine-induced status epilepticus (PISE)^8^. This study demonstrated dynamic changes in [^11^C]ABP688 availability for mGluR5 in the striatum and hippocampus. A PET study for mGluR1, another subtype of group I mGluRs, has never been performed in PISE rats because there was a lack of useful mGluR1 PET ligands allowing noninvasive quantification.

Recently, our research group has developed a useful new PET ligand to image mGluR1 called as *N*-[4-[6-(isopropylamino)-pyrimidin-4-yl]-1,3-thiazol-2-yl]-*N*-methyl-4-[^11^C]methylbenzamide ([^11^C]ITDM). This ligand has high bind affinity (Ki = 13.6 nM) and selectivity for mGluR1^9^. In addition, [^11^C]ITDM-PET allows quantification of slight changes in mGluR1 density in experimental animal models of CNS disorders, such as Huntington’s disease and Parkinson’s disease^10,11^.

In the present study, we aimed to monitor quantitative regional changes in mGluR1 density using longitudinal [^11^C]ITDM-PET in rats after PISE. We simultaneously evaluated neuroinflammation by PET with *N*-benzyl-*N*-[^11^C]methyl-2-(7-methyl-8-oxo-2-phenyl-7,8-dihydro-9H-purin-9-yl)acetamide ([^11^C]DAC), a selective PET ligand for translocator protein (TSPO)^12^, because repeated seizures can induce neuroinflammation following excess glutamate release^13^.

## Results

### PET studies for [^11^C]ITDM with mGluR1

**Figure 1:**
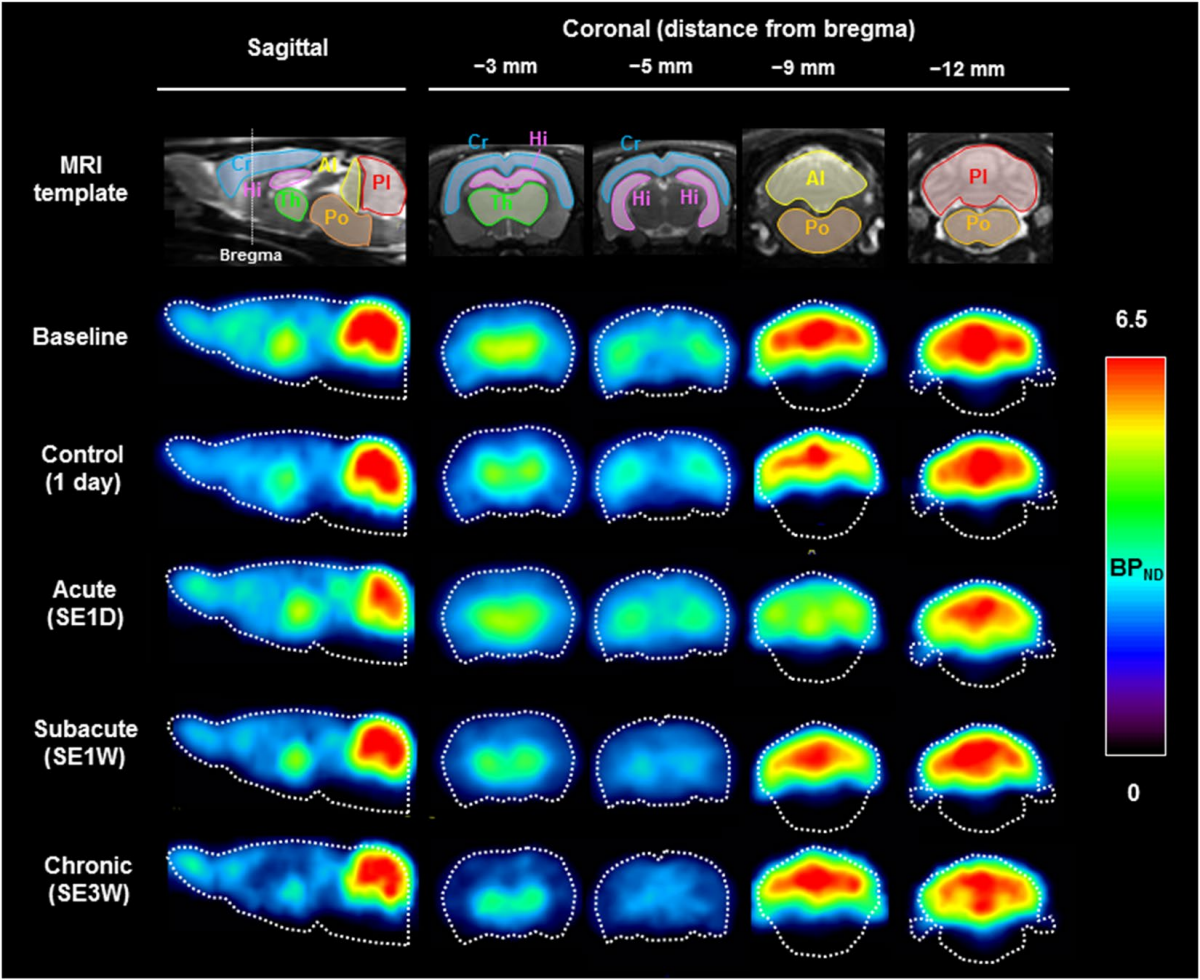
Magnetic resonance imaging (MRI) templates and representative parametric positron emission tomography (PET) images of BP_ND_ scaled [^11^C]ITDM in the rat brain after PISE. The tomograms were reconstructed in sagittal or coronal planes. Abbreviations: Cr, cerebral cortex; Hi, hippocampus; Th, thalamus; Al, anterior lobes of the cerebellum; Pl, posterior lobes of the cerebellum; Po, pons.

We estimated [^11^C]ITDM availability (BP_ND_) for mGluR1 in PISE rats (n = 6) on each experimental time points. Figure 1 shows representative parametric PET images based on the mGluR1-BP_ND_ scale for each experimental time point and Table 1 exhibits detail results of PET studies. At the acute period (1 day after status epilepticus (SE); SE1D), there was a substantial decline (2.83 ± 0.18) in signal compared with the baseline (4.07 ± 0.56, n = 4) and the control (4.41 ± 0.20, n = 4) levels in the coronal (−9 mm from bregma) images of the anterior lobes of the cerebellum. This decrease subsequently recovered to a normal level (4.12 ± 0.46) over the subacute period (1 week after SE; SE1W). Conversely, signals in sagittal and coronal sections of the thalamus (−3 mm from bregma) and hippocampus (−5 mm from bregma) decreased gradually across the chronic period (3 weeks after SE; SE3W). In the thalamus, there was no acute change (2.28 ± 0.17) compared with baseline BP_ND_ (2.30 ± 0.36). However, there was a subsequent significant decrease (*p* = 0.04) over the chronic period (1.78 ± 0.17).

**Table 1:** Regional changes of BP_ND_ for [^11^C]ITDM at the baseline, control, acute (SE1D), subacute (SE1W) and chronic (SE3W) time points.

Regions	BP_ND_ values (mean ± s.d.)				
	Baseline^a^	Control (post 1 day)^a^	Acute (SE1D)^b^	Subacute (SE1W)^b^	Chronic (SE3W)^b^
Cingulate cortex	1.54 ± 0.30	1.50 ± 0.20	1.53 ± 0.17	1.15 ± 0.18	1.27 ± 0.09
Cerebral cortex	0.80 ± 0.24	0.76 ± 0.07	0.91 ± 0.15	0.69 ± 0.07	0.99 ± 0.44
Striatum	1.92 ± 0.37	1.79 ± 0.05	1.92 ± 0.15	1.54 ± 0.22	1.69 ± 0.15
Thalamus	2.30 ± 0.36	2.20 ± 0.07	2.28 ± 0.17	1.82 ± 0.27	1.78 ± 0.17*
Hippocampus	1.67 ± 0.33	1.65 ± 0.13	1.58 ± 0.18	1.25 ± 0.21	1.32 ± 0.16
Anterior lobes of cerebellum	4.07 ± 0.56	4.41 ± 0.20	2.83 ± 0.18***	4.12 ± 0.46	4.36 ± 0.23
Posterior lobes of cerebellum	4.32 ± 0.60	4.67 ± 0.12	3.86 ± 0.40	4.45 ± 0.51	4.64 ± 0.49

^a^n = 4; ^b^n = 6.

*P < 0.05, ***P < 0.001 (two-way analysis of variance with post hoc tests, compared with the baseline group).

### ***In vitro*** autoradiography for mGluR1 with [^11^C]ITDM

**Figure 2:**
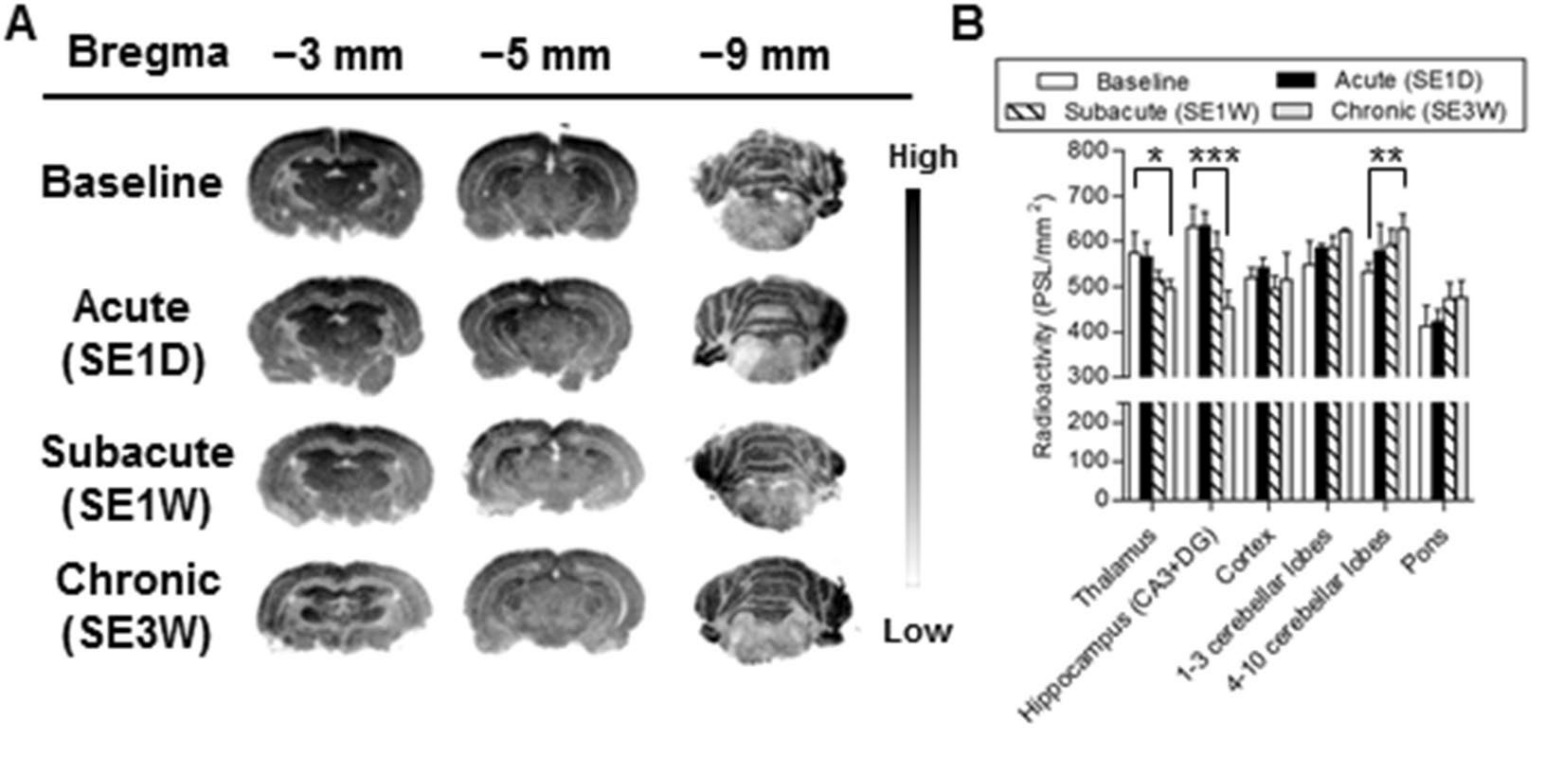
Representative *in vitro* autoradiograms (**A**) and quantitative values (**B**) of specific binding of [^11^C]ITDM in coronal brain slices prepared from rats at the control, acute (SE1D), subacute (SE1W) and chronic (SE3W) time points.

To validate the changes observed in mGluR1-BP_ND_ during *in vivo* PET, we conducted *in vitro* autoradiography using brain sections from rats after PISE. Figure 2A shows representative *in vitro* [^11^C]ITDM autoradiograms using coronal brain sections prepared at different distances from bregma (−3, −5 and −9 mm) in rats after PISE. There was a significant decline in radioactivity in the thalamus and hippocampus (CA3 and dentate gyrus) after the acute period, which corroborated the PET results. In particular, the largest decline in the thalamus was observed during the chronic period, with an approximately 30% decrease compared with control sections (Fig. 2B). There was no significant decrease in radioactive signals in the anterior lobes of cerebellum (−9 mm coronal section) in the acute period, conflicting with results from the corresponding PET images. There was also no significant difference between rats sacrificed at baseline and the acute time point in quantitative *in vitro* [^11^C]ITDM binding values. Conversely, there was a gradual increase of radioactivity in the cerebellar lobes with time after PISE (Fig. 2B).

### PET imaging for neuroinflammation

**Figure 3:**
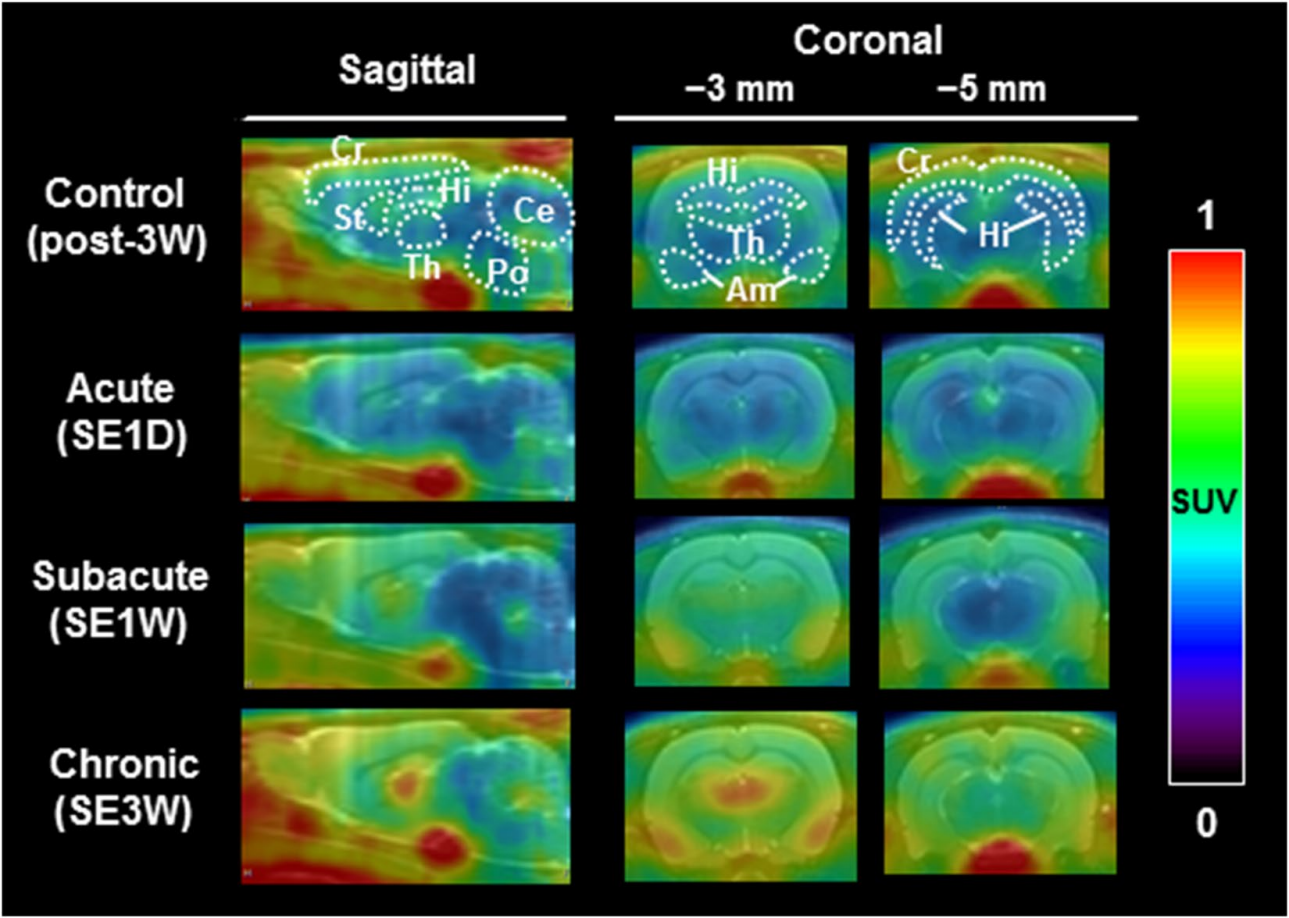
Representative averaged PET/MRI images of [^11^C]DAC in the rat brain of control (3 weeks after diazepam treatment), acute (1 day after SE), subacute (1 week after SE) and chronic (3 weeks after SE) subjects. The images show sagittal and coronal views. Radioactivity was expressed by SUV. Abbreviations: Cr, cerebral cortex; St, striatum; Hi, hippocampus; Th, thalamus; Am, amygdala; Ce, cerebellum; Po, pons.

To estimate neuroinflammation in the PISE model, TSPO-PET imaging with [^11^C]DAC was conducted on PISE rats (n = 4). Figure 3 shows representative averaged PET images of [^11^C]DAC in the brains of control, acute, subacute and chronic subjects. At the acute period, radioactive uptake of [^11^C]DAC was low across the whole brain, similar to that of control. The area under the curve (AUC) values (SUV × min) of control and acute animals were 17–23 and 16–23, respectively (Table 2). In contrast, an increase of radioactive uptake of [^11^C]DAC was observed in the forebrain (cerebral cortex, striatum, thalamus, hippocampus and amygdala) of subacute and chronic animals. A substantial increase in the AUC value was observed in the amygdala for subacute (AUC 38) and chronic (AUC 39) animals. Significant differences (*p* < 0.001) compared with control animals were found in the cerebral cortex, striatum, thalamus and hippocampus for both subacute and chronic animals. Radioactive uptake of [^11^C]DAC in the cerebellum and pons indicated no differences at any time points.

**Table 2:** Regional changes of area under the curve (AUC) values (SUV × min) for [^11^C]DAC at the control, acute (SE1D), subacute (SE1W) and chronic (SE3W) time points.

Regions	AUC values (mean ± s.d.)			
	Control (post 3 weeks)^a^	Acute (SE1D)^b^	Subacute (SE1W)^b^	Chronic (SE3W)^b^
Cerebral cortex	21.8 ± 1.8	18.8 ± 0.8	29.3 ± 2.4***	29.7 ± 1.8***
Striatum	17.0 ± 1.6	16.0 ± 0.8	27.4 ± 1.4 ***	26.3 ± 2.8***
Thalamus	17.7 ± 1.1	15.6 ± 0.5	25.8 ± 2.1***	29.3 ± 3.3***
Hippocampus	17.1 ± 1.1	16.9 ± 0.8	28.0 ± 2.2***	28.0 ± 2.3***
Amygdala	19.7 ± 1.4	22.6 ± 2.2	38.6 ± 4.8***	39.3 ± 5.8***
Cerebellum	23.2 ± 3.5	19.7 ± 0.5	21.9 ± 1.4	24.8 ± 1.9
Pons	23.4 ± 3.2	22.2 ± 0.8	22.5 ± 0.4	23.7 ± 2.4

^a^n = 3; ^b^n = 4.

***P < 0.001 (two-way analysis of variance with post hoc tests, compared with the control group).

### *In vitro* binding of [^11^C]DAC and TSPO localization

**Figure 4:**
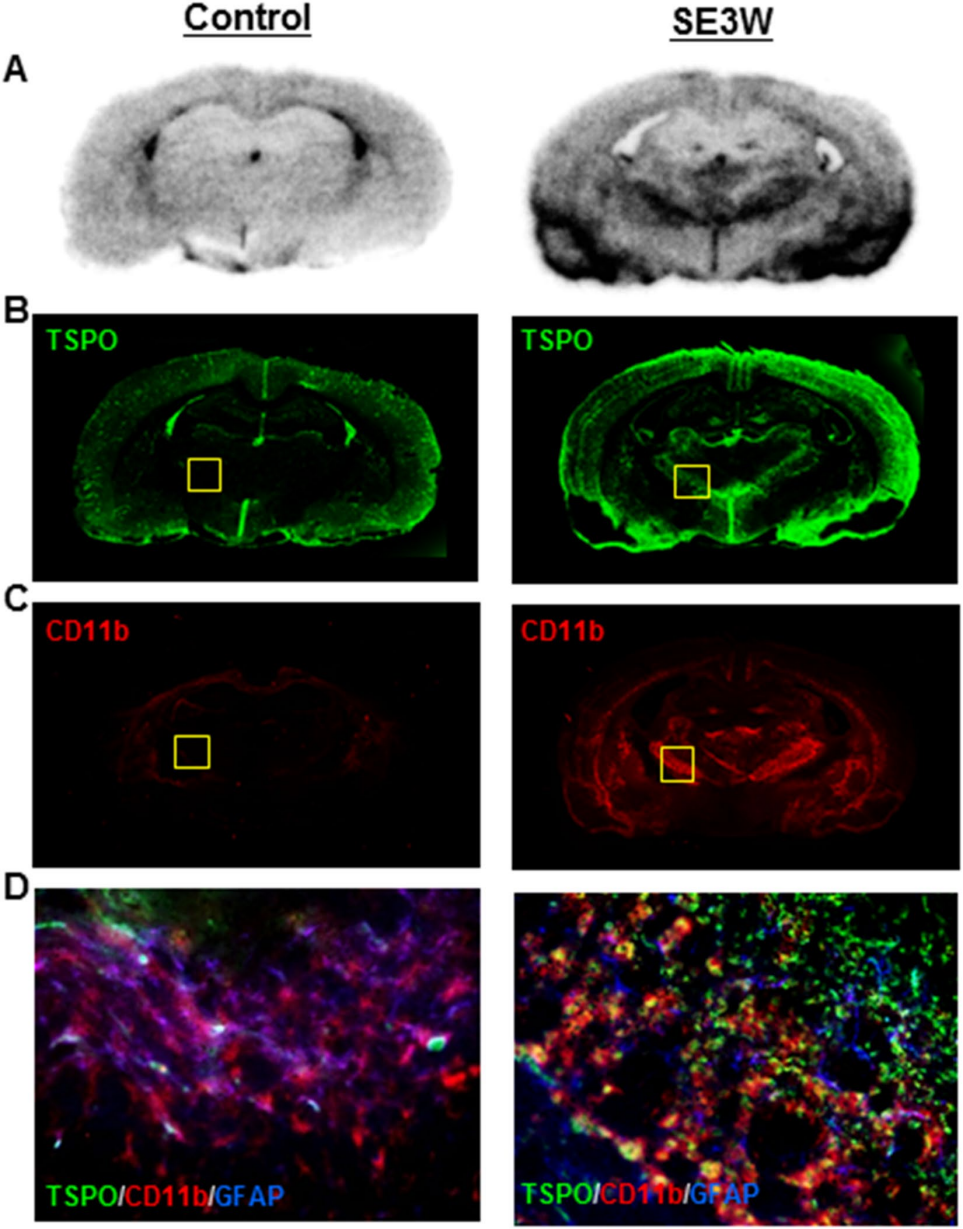
Representative *in vitro* autoradiograms (**A**) of [^11^C]DAC and immunohistochemical images for TSPO (**B**), CD11b (**C**) and TSPO/CD11b/GFAP (**D**). Brain sections were prepared from the control (left column) and chronic (right column) animals.

TSPO is expressed not only in activated microglial cells, but also in cerebral blood vessels^14^. To confirm whether brain uptake of [^11^C]DAC in the PET analysis corresponds with TSPO expression in activated microglial cells, *in vitro* autoradiography with [^11^C]DAC and immunohistochemical images for TSPO were compared. Furthermore, pathological evaluation for microglial activation was conducted using an anti-CD11b antibody. Figure 4 shows representative *in vitro* autoradiograms of [^11^C]DAC and immunohistochemical images of brain sections removed from control and chronic animals. As shown in the PET result, the autoradiogram of chronic animals also exhibited highly radioactive accumulations of [^11^C]DAC in the thalamus and around the amygdala (Fig. 4A). Immunohistochemistry for TSPO detected relatively high fluorescent signals on the cerebral cortex, thalamus and around the amygdala, which partly corresponded with distribution of radioactivity in autoradiograms, with the exception of the cerebral cortex (Fig. 4B). Because the cerebral cortex of control sections exhibited high signals, cerebral neocortical signals in both groups likely indicate TSPO in the blood vessels. Immunohistochemical images for CD11b detected high signals in the thalamus and around the amygdala (Fig. 4C). Colocalization with CD11b was detected with half of the TSPO expression in the thalamus of chronic animals, whereas TSPO signals were not detected with glial fibrillary acidic protein (GFAP) signals indicating astrocyte localization (Fig. 4D). Taken together, [^11^C]DAC may exhibit specific binding to TSPO on activated microglial cells but not vascular TSPO.

### Comparison between [^11^C]ITDM availabilities and [^11^C]DAC accumulations

**Figure 5:**
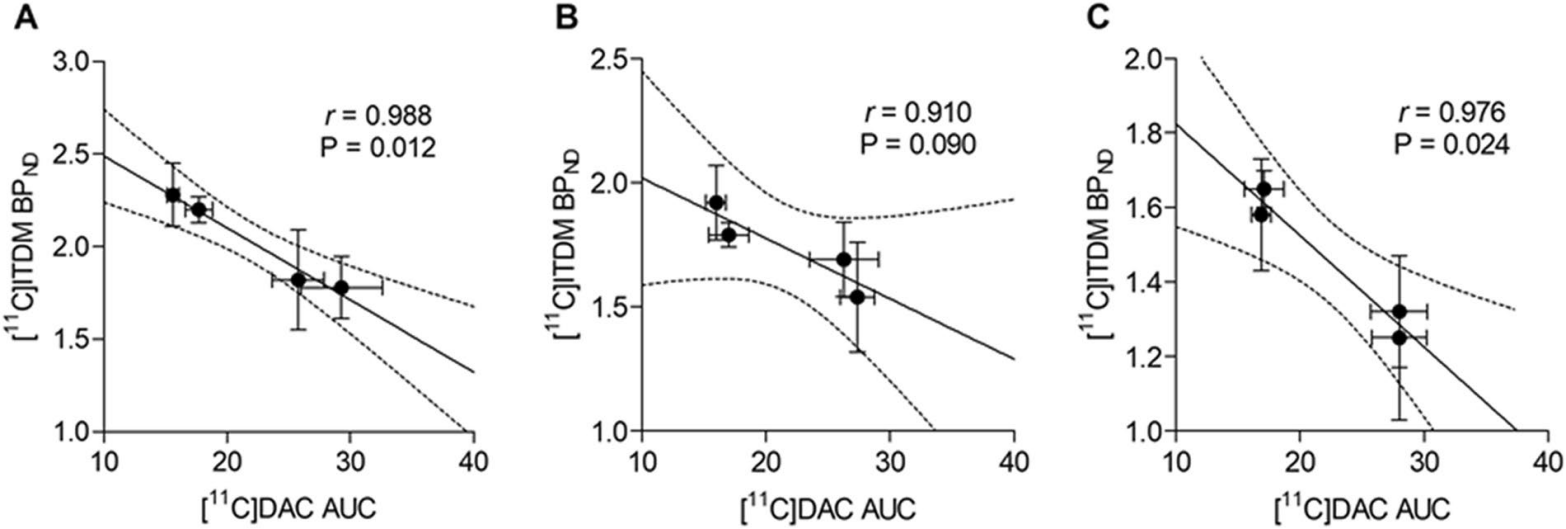
Correlation between BP_ND_s for mGluR1 with [^11^C]ITDM and AUCs for TSPO with [^11^]DAC in the thalamus (**A)**, striatum (**B**) and hippocampus (**C**). The regression lines in each graph show the 95% confidence intervals (dotted lines). Respective correlation coefficients (*r*) and P values are cited adjacent to each scatter plot.

To confirm the correlation between the decline of [^11^C]ITDM with mGluR1 and microglial activation, a correlational plot analysis was performed. Figure 5 shows the scatter plot between [^11^C]ITDM availability (BP_ND_) and [^11^C]DAC uptake (AUC) in the thalamus, striatum and hippocampus at each time point. The BP_ND_s of [^11^C]ITDM indicated a negative correlation with AUC values of [^11^C]DAC in all brain regions. The correlation coefficients (*r*) and P values in the thalamus, striatum and hippocampus were 0.988 and 0.012, 0.910 and 0.090, and 0.976 and 0.024, respectively. These statistical results could support high correlation between the two parameters.

## Discussion

Previously, it has been reported that immunoreactivity for hippocampal mGluR1α, which is one of three mGluR1 splice variants, shows a marked decrease 1 day after PISE, and that mGluR1α-immunoreactive dendrites and cell bodies gradually reappear between 3 and 31 days after PISE^15^. However, in this study, PET and autoradiography using [^11^C]ITDM did not detect a hippocampal decline of specific binding with mGluR1 at the acute period after PISE. In contrast, *in vitro* and *in vivo* specific binding of [^11^C]ITDM with mGluR1 showed a gradual decline between 1 and 3 weeks after PISE (Figs 1 and 2). The mGluR1β variant has a different carboxy-terminal from mGluR1α, and is strongly identified in principal cells of CA3 and dentate granule cells, but is absent in CA1^16^. *In vitro* autoradiography with [^11^C]ITDM showed high radioactive accumulations in the CA3 and dentate gyrus areas but not in the CA1 area (Fig. 2). Therefore, [^11^C]ITDM availability may reflect specific binding with mGluR1β but not mGluR1α.

In another report, astrocytes expressing several types of protein kinase C related to gliosis have been identified in all parts of the hippocampus at 7 to 31 days after PISE^17^. Thus, we further evaluated microglial activation using a longitudinal PET study with [^11^C]DAC, a second generation TSPO PET ligand. TSPO is a biomarker for activated microglia^18^. In the present PET analysis with [^11^C]DAC, high radioactive uptake was detected in the forebrain of rats at the subacute period, maintaining a high level until the chronic period. Taken together, these results suggest that gradual decline of [^11^C]ITDM availability in the hippocampus may reflect disappearance of mGluR1β on neuronal cells caused by gliosis, though there has been no pathological evidence for mGluR1β until now.

Remarkable levels of radioactive uptake of [^11^C]DAC were observed around the amygdala and in the thalamus, which corresponds with localization of glutamatergic principal neurons. Additionally, pathological evaluation showed TSPO/CD11b colocalization in the ventral posterolateral thalamic nuclei (Fig. 4E). These deposits corresponded with the region demonstrating a remarkable decline of [^11^C]ITDM availability, which further indicates a negative correlation with accumulation of [^11^C]DAC (Fig. 5). These findings indicate that neuroexcitotoxicity induced by repeated seizures stimulates microglial activation, subsequently inducing injury to glutamatergic principal neurons.

In present study, the most severe decline in [^11^C]ITDM availability with mGluR1 was found in the anterior lobes of the cerebellum over the acute period of PISE (Fig. 1). Surprisingly, results from quantitative autoradiography using [^11^C]ITDM showed no change in the *in vitro* binding in the anterior lobes of the cerebellum between pre- and post-SE. Inconsistencies between *in vitro* autoradiography and *in vivo* PET results may be caused by regional differences in blood flow. In a previous report using [^11^C]verapamil-PET in a PISE model, it was demonstrated that cerebral blood flow in the cerebellum significantly decreased in the acute period^19^. Actually, parameter R1, the ratio (K1/K1’) of tracer delivery in simplified reference tissue model (SRTM)^20^, in the cerebellum showed a transient reduction in the anterior lobes at acute period (see Supplementary Fig. S1). As such, large decreases in availability of [^11^C]ITDM in the anterior lobes of rats after PISE in the acute period may be caused by cerebral blood flow decreases, which then recover over the subacute period. The mechanisms by which this might occur are unknown.

A pilot PET study using [^11^C]ABP688 has demonstrated a temporal decrease of availability for mGluR5 in the striatum and hippocampus at the acute period of the PISE model and subsequent recovery over the chronic period^8^. Interestingly, the present PET study with [^11^C]ITDM exhibited unchanged availability in the striatum and a gradual decline of availability in the thalamus and hippocampus across the chronic period. Based on these findings, we hypothesize that in acute period of PISE, striatal and hippocampal mGluR5 and mGluR1α, which may not bind with [^11^C]ITDM, may be downregulated to protect against acute neuroexcitotoxicity, but not [^11^C]ITDM with mGluR1. Subsequently, their temporal downregulations gradually recover. In contrast, neuroinflammation gradually occurs in the forebrain after the acute period of PISE. Incidentally, [^11^C]ITDM with mGluR1 may gradually disappear in CA3, dentate gyrus and ventral posterolateral thalamic nuclei. Taken together, these findings suggest that repeated seizures stimulate glutamatergic neurons and subsequently induce different genetic regulation of mGluRs belonging to group I, and result in neuroinflammation caused by gliosis.

In conclusion, we visualized heterogeneous changes in [^11^C]ITDM availability with mGluR1 in rat brains after PISE via longitudinal PET studies. In particular, we observed a gradual decrease of [^11^C]ITDM availability in the thalamus and hippocampus. Moreover, we showed that their reductions were involved in neuronal injury caused by microglial activation. Thus, molecular imaging with [^11^C]ITDM will be a useful tool for gaining further understanding of the mechanisms leading to neurotransmitter dysfunction and aid the development of new pharmaceuticals for CNS disorders.

## Materials and Methods

### Generals

We purchased all chemicals used in this study from commercial companies, including Sigma-Aldrich (St. Louis, MO, USA), Wako Pure Chemicals Industries (Osaka, Japan) and Tokyo Chemical Industries (Tokyo, Japan). We prepared diazepam hydrochloride in-house from diazepam (sourced from Wako Pure Chemicals Industries).

Male Sprague–Dawley rats were purchased from Japan SLC and kept in a temperature-controlled environment with a 12-h light/dark cycle. They were given a standard diet (MB-1, Funabashi Farm) and water containing hyper diluted hydrochloric acid.

### Ethics statement

The rats were treated and handled according to the Recommendations for Handling of Laboratory Animals for Biomedical Research, compiled by the Committee on the Safety and Ethical Handling Regulations for Laboratory Animal Experiments of the National Institutes for Quantum and Radiological Science and Technology, and this study was approved by the committee.

### Radiochemistry

[^11^C]ITDM was synthesized via the reaction of a stannyl precursor with [^11^C]methyl iodide in the presence of triphenylphosphine and copper chloride as described previously^9^. At the end of synthesis, [^11^C]ITDM was obtained with >99% radiochemical purity and 167–370 GBq/µmol (n = 10) specific activity.

[^11^C]DAC was synthesized by reacting desmethyl precursor with [^11^C]methyl iodide in the presence of sodium hydroxide as described previously^12^. At the end of synthesis, [^11^C]DAC was obtained with >98% radiochemical purity and 30–111 GBq/µmol (n = 10) specific activity.

### Production of PISE model

Rats with PISE were produced as described previously^8^. Briefly, male rats (n = 32) were intraperitoneally injected with lithium chloride (127 mg/kg) and were then intraperitoneally administered methylscopolamine bromide (1 mg/kg) after 20–24 h. After another 30 min, pilocarpine hydrochloride (30 mg/kg) was intraperitoneally injected to trigger SE. Pilocarpine hydrochloride (10 mg/kg) was then repeatedly administered (2–4 times) every 30 min until the appearance of stage 4 seizures (according to the Racine scale)^21^. The rats expressing repeated seizures (n = 28) were intraperitoneally administered diazepam hydrochloride (10 mg/kg) 60 min after the onset of SE to terminate seizure activity. Diazepam hydrochloride (5 mg/kg) was repeatedly administered until SE was terminated to reduce mortality (1–3 times). Control rats were administered the same drugs except pilocarpine.

### Small-animal PET procedure

PET scans using the same PISE rat were conducted at 1 day (SE1D), 1 week (SE1W) and 3 weeks (SE3W) after PISE and categorized as acute, subacute and chronic periods, respectively. Each rat was anesthetized with 1.5% (v/v) isoflurane and a 24-gauge intravenous catheter (Terumo Medical Products, Tokyo, Japan) was inserted into the tail vein. The rat was subsequently maintained under anaesthesia and secured in a custom-designed chamber placed in the centre of a small-animal PET scanner (Inveon; Siemens Medical Solutions, Knoxville, TN, USA). After adjustment to ensure target positioning for brain scanning, the dynamic emission scans (in three-dimensional list mode) were performed for 90 min (10 s × 12 frames, 20 s × 3 frames, 30 s × 3 frames, 1 min × 3 frames, 2.5 min × 3 frames and 5 min × 15 frames) during which [^11^C]ITDM was administered (48–60 MBq, 0.1–0.3 nmol) via the tail vein catheter. For [^11^C]DAC (25–60 MBq, 0.1–0.4 nmol), dynamic emission scans were carried out for 60 min (10 s × 12 frames, 20 s × 3 frames, 30 s × 3 frames, 1 min × 3 frames, 2.5 min × 3 frames and 5 min × 9 frames). Throughout the scan, the rat’s body temperature was maintained at 37 °C using a heated (40 °C) water circulation system (T/Pump TP401; Gaymar Industries, Orchard Park, NY, USA). After the PET experiments, rats were allowed to recover from anaesthesia and were returned to the animal breeding facility. Note: [^11^C]ITDM- and [^11^C]DAC-PET studies were conducted using different PISE rats.

### Data analysis

The tissue time–activity curve (tTAC) was acquired from the volume of interest (VOI). VOIs for [^11^C]ITDM were anatomically mapped onto the cingulate cortex, cerebral cortex, striatum, hippocampus, thalamus, anterior and posterior lobes of the cerebellum and the pons. VOIs for [^11^C]DAC were located in the cerebral cortex, striatum, thalamus, hippocampus, amygdala, cerebellum and pons. The radioactivity was expressed as the standardized uptake value (SUV), which was normalized to the injected radioactivity and bodyweight. The SUV was calculated according to the following formula: SUV = (radioactivity per milliliter tissue/injected radioactivity) × bodyweight (g). To obtain the availability (BP_ND_) of [^11^C]ITDM, kinetic analyses were performed using the Logan reference method^22^. In accordance with our previous reports^10,23^, Logan reference analysis was performed by linear regression, with t* = 15 min and using tTACs obtained from the pons as a reference region. For [^11^C]DAC-PET, the quantitative value for TSPO was expressed as the AUC (SUV × min) during 2–60 min after the injection, because there was a lack of reference region for TSPO.

### *In vitro* autoradiography

The PISE rat different from PET assessments use was anesthetized using 1.5% (v/v) isoflurane and sacrificed by cervical dislocation. Their brains were quickly removed and frozen. The frozen brains were cut into sections of 20 µm using a cryostat (HM560; Carl Zeiss, Oberkochen, Germany), and the slices were mounted on glass slides (Matsunami Glass, Tokyo, Japan). The brain sections were preincubated at room temperature for 20 min in 50 mM Tris-HCl buffer (pH 7.4), containing 1.2 mM MgCl_2_ and 2 mM CaCl_2_. The sections were then incubated for 30 min at room temperature in fresh buffer containing [^11^C]ITDM (7.4 MBq, 0.1 nM) or [^11^C]DAC (7.4 MBq, 0.7 nM). After incubation, the brain sections were washed (3 min × 3 times) with cold buffer, dipped in cold distilled water and air-dried. They were then placed in contact with imaging plates (BAS-MS2025; Fujifilm, Tokyo, Japan). Autoradiograms were acquired using a Bio-Imaging Analyzer System (BAS5000; Fujifilm). The degree of binding (PSL/mm^2^) was calculated using Multi Gauge analysis software version 2.3 (Fujifilm).

### Immunohistochemistry

Immunohistochemically examinations were conducted using the same brain sections used for autoradiography with [^11^C]DAC. The sections were soaked in hexane for 10 min at room temperature, subsequently fixed with 4% paraformaldehyde, and then washed with phosphate-buffered saline. Respective primary antibody incubations were performed using a rabbit anti-mouse TSPO antibody (NP155, 1:1000)^24^, a monoclonal mouse anti-rat CD11b antibody (1:100, AbD Serotec) and a rat anti-GFAP antibody (1:500, Thermo Fisher Scientific). The sections were incubated with the primary antibodies overnight at 4 °C. After the first immunoreaction, the sections were incubated with Alexa 647-conjugated anti-mouse IgG secondary antibody (1:500; Thermo Fisher Scientific), Alexa 546-conjugated anti-rat IgG antibody (1:500; Thermo Fisher Scientific) and biotin-conjugated anti-rabbit IgG secondary antibody (1:1000; Thermo Fisher Scientific) for 1 h at room temperature, followed by tyramide signal amplification using a Fluorescein System (FITC) (PerkinElmer, Waltham). The sections were washed with phosphate-buffered saline and mounted with medium (Vector Laboratories). Fluorescent images were captured using a fluorescence microscope (BZ-9000, Keyence).

### Statistical analysis

All data are expressed as mean ± standard deviation (s.d.). Differences between baseline and after PISE were calculated using two-way repeated measures analysis of variance. Post hoc analyses with Bonferroni methods were applied. Statistical significance (denoted with asterisks on the figures) was determined at a 95%, P < 0.05, confidence level. All statistical data were analyzed using GraphPad Prism 5 (GraphPad Software).

## Electronic supplementary material

Figure S1
