# Supplementary material for: In Vivo Monitoring for Regional Changes of Metabotropic Glutamate Receptor Subtype 1 (mGluR1) in Pilocarpine-Induced Epileptic Rat Brain by Small-Animal PET
Source: Sci Rep. 2017 Nov 2;7:14945. doi: 10.1038/s41598-017-15015-2 (PMC5668420; doi:10.1038/s41598-017-15015-2)
Supplement: Supplementary file 1 — Figure S1 [file 41598_2017_15015_MOESM1_ESM.pdf]

In Vivo Monitoring for Regional Changes of Metabotropic Glutamate Receptor Subtype 1 (mGluR1) in Pilocarpine-Induced Epileptic Rat Brain by Small-Animal PET

Tomoteru Yamasaki, Masayuki Fujinaga, Wakana Mori, Yiding Zhang, Hidekatsu Wakizaka, Nobuki Nengaki, Lin Xie, Akiko Hatori & Ming-Rong Zhang

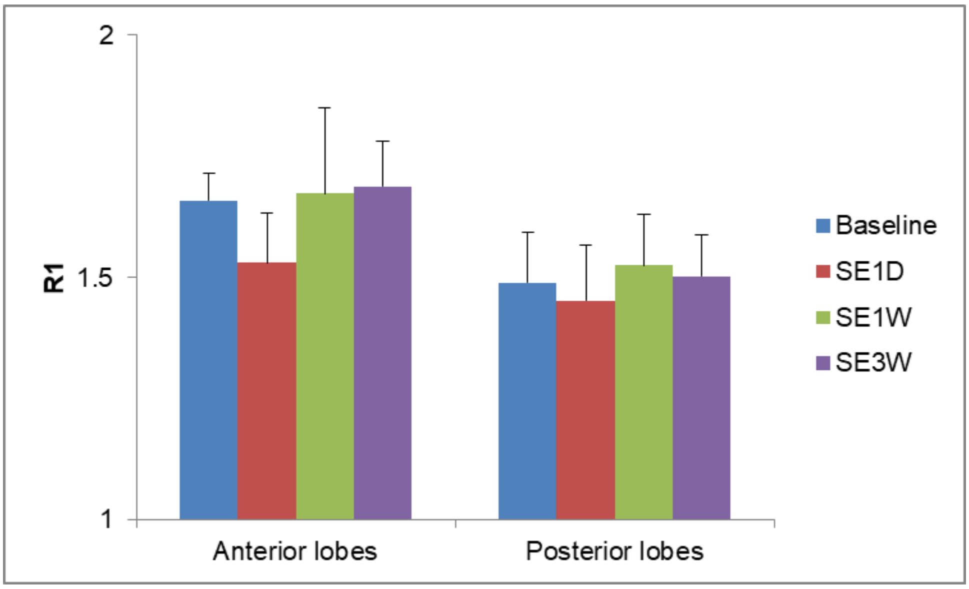

Fig. S1 Parameter R1 based on simplified reference tissue model (SRTM) in the anterior lobes and posterior lobes of cerebellum. At acute period (SE1D), R1 in the anterior lobes of cerebellum indicated slight reduction, which suggested decline of input function K1 of anterior lobes at acute period.
